# A new geospatial overlay method for the analysis and visualization of spatial change patterns using object-oriented data modeling concepts

**DOI:** 10.1080/15230406.2014.901900

**Published:** 2014-03-28

**Authors:** Dirk Tiede

**Affiliations:** ^a^ Department of Geoinformatics - Z_GIS, University of Salzburg, Schillerstr. 30, 5020Salzburg, Austria

**Keywords:** object-based change analysis, topological, change comparison, object-oriented data modeling

## Abstract

Traditional geographic information system (GIS)-overlay routines usually build on relatively simple data models. Topology is – if at all – calculated on the fly for very specific tasks only. If, for example, a change comparison is conducted between two or more polygon layers, the result leads mostly to a complete and also very complex from–to class intersection. A lot of additional processing steps need to be performed to arrive at aggregated and meaningful results. To overcome this problem a new, automated geospatial overlay method in a topologically enabled (multi-scale) framework is presented. The implementation works with polygon and raster layers and uses a multi-scale vector/raster data model developed in the object-based image analysis software eCognition (Trimble Geospatial Imaging, Munich, Germany). Advantages are the use of the software inherent topological relationships in an object-by-object comparison, addressing some of the basic concepts of object-oriented data modeling such as classification, generalization, and aggregation. Results can easily be aggregated to a change-detection layer; change dependencies and the definition of different change classes are interactively possible through the use of a class hierarchy and its inheritance (parent–child class relationships). Implementation is exemplarily shown for a change comparison of CORINE Land Cover data sets. The result is a flexible and transferable solution which is – if parameterized once – fully automated.

## Introduction and problem definition

Many change-analysis approaches are documented in the literature – see for example, the overview articles from Coppin et al. (2004), Lu et al. (2004), Singh (1989), and Chen et al. (2012). Some of this research deals with post-classification methods, often termed as post-classification comparison (PCC). This paper focuses on PCCs based on classification results from remotely sensed imagery of different time stamps, but, in principle, it addresses change comparisons of any geospatial classification data sets. For such comparisons, pixel-by-pixel or object-by-object comparisons are usually conducted. Traditional geographic information system (GIS) overlay or intersect routines induce complete (and complex) matrices of change, which results in specific challenges such as generation of binary change images with no recognition of major and minor landscape changes, or a complex class-by-class-based evaluation (Ahlqvist 2008). Many processing steps have to be performed to reach aggregated and – what is often needed – more high-level results of changes. Even if these steps are established as repeatable models or protocols, the analysis is limited by the restriction of relational data models in most GIS (cf. De Smith et al. 2007; Longley et al. 2005).

In this study, I make use of another approach which is theoretically formulated but not available in conventional GIS (cf. Chen, Wang, and Chen 2013): Egenhofer and Frank (1992) presented in a survey article concepts of object-oriented modeling applied to geographic data. Based on the original work by Brodie (1984), they described how an object-oriented data model is built on the four basic concepts of abstraction, namely classification, generalization, association, and aggregation. Based on these concepts, a comparison of different data sets (i.e., different classifications such as land use/land cover data sets) would theoretically overcome the aforementioned problems. The following selected concepts of object-oriented data modeling are taken from Egenhofer and Frank (1992) and are examined for their potential to improve the overlay processing in PCCs:

- Concept of generalization: Generalization in object-oriented modeling should not be confused with the same term used in cartography. In the former, it expresses the idea of grouping several classes of objects with common properties or operations into a more general superclass. For a PCC, such a super-class could represent a “change class” that has subsumed certain changes of interest (e.g., if the land-cover classes forest or scrubland are changing into bare soil or grassland, the concept of generalization can help to define super-classes such as “degradation”, “urban sprawl,” or similar). Such a concept of generalization needs to be flexible, that is, the subsuming of classes to a superclass should be easily changed if needed.
- Concept of association: Relationships between objects, also called grouping or partitioning. According to Egenhofer and Frank (1992), an example of an association in the GIS domain is neighborhood (e.g., there may be a relationship between a land parcel and an adjacent house lot). In the presented case of PCC, it could also be a relationship among objects through time, for example, a homogeneous land cover object is divided on the second time stamp into several objects (different classes) but the objects are still related and can be accessed (e.g., through a query).
- Concept of aggregation: Similar to association, aggregation is the combination of objects to form a higher level object (in the semantic sense). Egenhofer and Frank (1992) termed this higher level object an aggregate or composite object, with the aggregated parts keeping their own functionality. An example would be the aggregation of changes to a higher level information object. Such an object could be an artificial unit (for instance, a regular gridded or hexagon layer), and also an administrative unit where the temporal changes of the aggregated objects are “reported”.

In the following chapter, I present a methodology which integrates the mentioned concepts of object-oriented data modeling to improve and simplify the overlay processes.

## Object-oriented geospatial overlay

The topologically enabled multi-scale data model proposed in this paper integrates the concepts of object-oriented data modeling presented earlier in order to automate the comparison of polygon (or raster) layers. The data model is implemented in a software package called eCognition (Trimble Geospatial Imaging), usually used in remote sensing applications for object-based image analysis (OBIA, see, e.g., Blaschke 2010).

**Figure 1.**
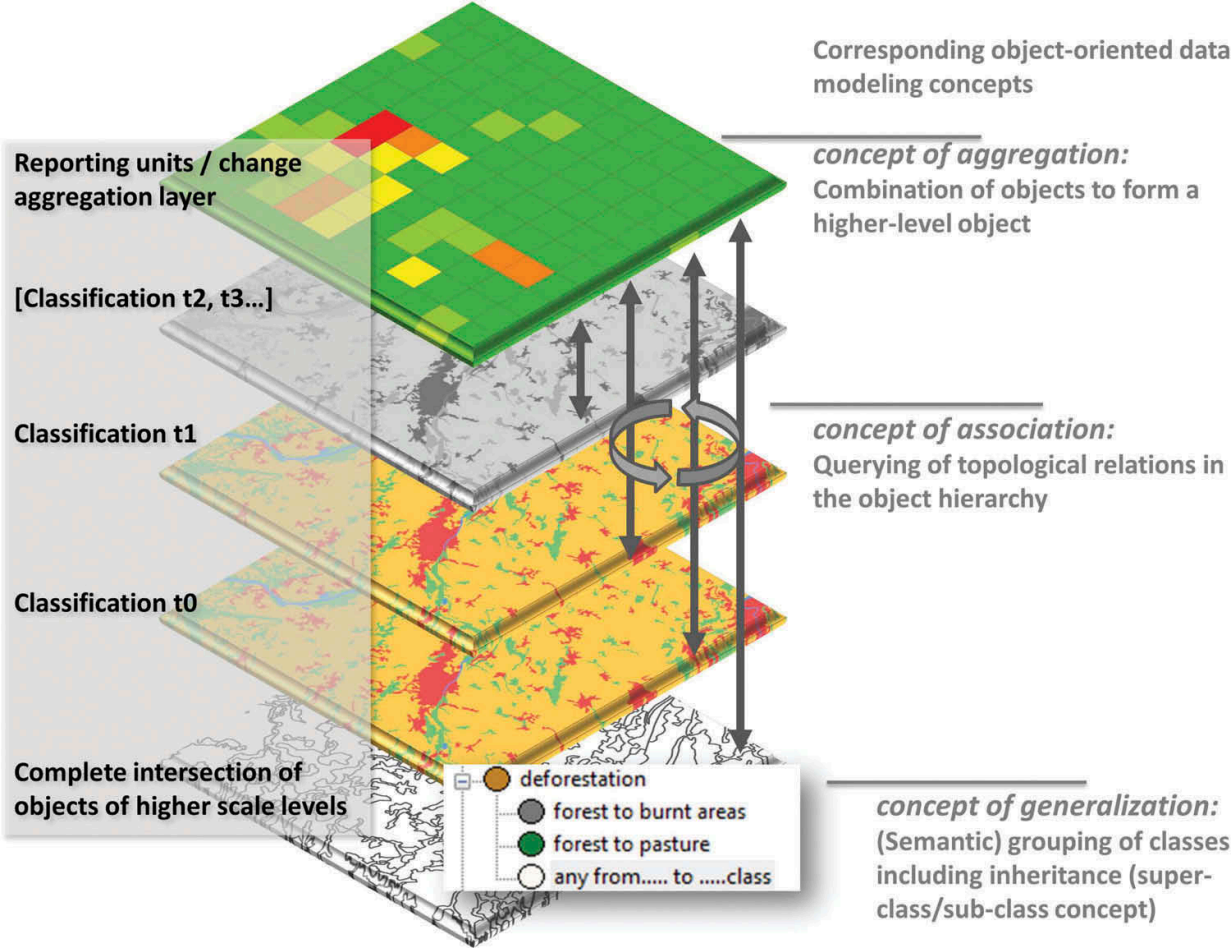
Schematic overview of the elements of the data model used in the context of PCC and the corresponding object-oriented data modeling concepts. The hierarchy of classification layers to be compared is not limited, and the horizontal and vertical topology makes it possible to query any relationship between objects/layers, based on classes and (semantic) groups of classes. Aggregation of changes to higher level objects or reporting units is possible.

The implemented data model is based on vector objects (usually built from image segmentation) and embedded in a hierarchy. Hierarchy in this context means that layers of different scales (if multi-scale segmentations are performed) are sorted in an increasing (scale-wise) order from bottom to top. It is a strict hierarchy; object (polygon) borders on a higher scale will usually be present at lower scales. This leads to a complete intersection of objects on the lowest scale if, for example, different classification layers are imported (cf. Figure 1). This data model comes from the image-analysis domain; the idea behind it, in short, is to mimic the human perception of images by looking and analyzing images at different (segmentation) scales (i.e., different “windows of perception”; Marceau 1999).

The implemented object-model forces a calculation of a complete topology during the calculation of each level or, in this case, during the import of externally available GIS layers. The topology encompasses not only a horizontal topology (between objects in one level) but also a vertical topology between the objects in the hierarchy. For each object, the whole levels and the hierarchy, so-called “features” are calculated. The term “features” originates from the image-analysis background of the software, but it can also be translated into GIS language: Features are the properties of each polygon and include, among other things, polygon-centered features such as size, form/shape descriptors, and classification-based statistics and also topological calculations such as the relation to neighboring objects (close by, but also in a certain distance), relational and absolute border share with neighbors, etc. These horizontal topological features are complemented by vertical ones describing the object within the (scale or temporal) hierarchy, which is a concept not available, to the author’s knowledge, in any GIS software or GIS data model in this consistency. Vertical topological features encompass such things as number of objects in lower levels covering the higher level objects, spatial relations between objects on different levels, and classifications in lower levels overlapping with higher level classifications and vice versa. Many of these features are calculated automatically when implementing the hierarchy; additional relationships between objects can be calculated, as needed. These topological features/relationships are saved in the data model and are available for further calculations.

In addition, the data model supports classes and class hierarchies, including an inheritance concept and grouping functionality. Figure 1 shows a schematic overview of the concept and how the different elements are corresponding to some of the object-oriented data modeling concepts presented by Egenhofer and Frank (1992). A more detailed description follows in the “Implementation” section.

Object hierarchy is used in this research to hold GIS layers representing classifications of different time stamps, not levels of different scales extracted from image layers. Instead of remotely sensed images, the data model is used to import GIS polygon layers (also raster would be possible) and perform change comparison analyses (geospatial overlay) by making use of the described advantages.

## Implementation example

**Figure 2.**
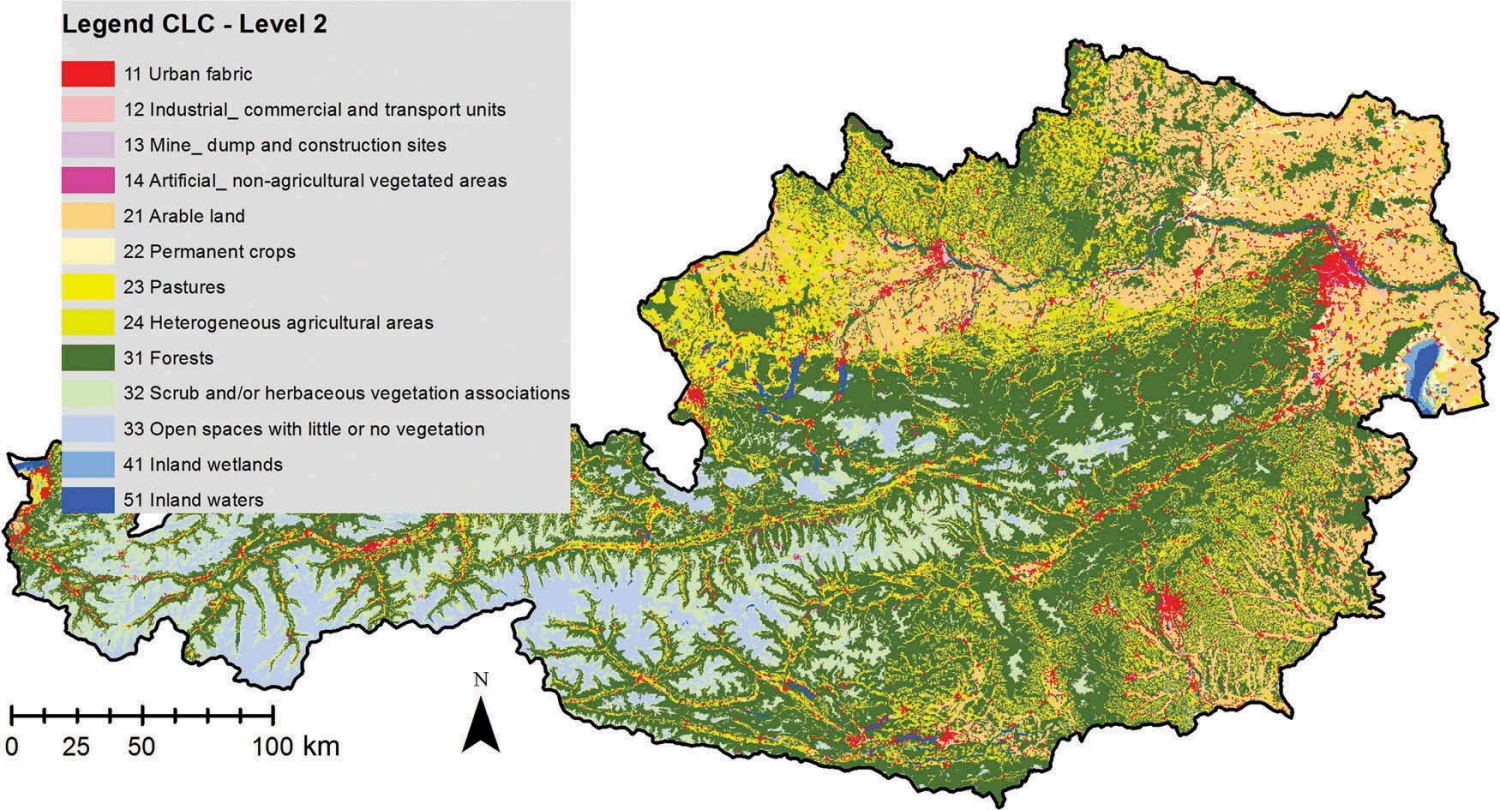
CLC for Austria. Visualized is the level two of the mapping hierarchy which comprises 13 subclasses. The first digit of the numbering indicates the category of the superclass (level one) to which the subclass belongs.

The implementation of the method is demonstrated using existing CORINE Land Cover (CLC) data sets from 2000 and 2006 as input data for the PCC and already existing CLC change data, based on expert interpretations, is used for evaluation. The standard CLC nomenclature includes 44 land cover classes (see Büttner and Kosztra 2007). These are grouped in a three-level hierarchy. The five main (level one) categories are: 1. artificial surfaces, 2. agricultural areas, 3. forests and seminatural areas, 4. wetlands, and 5. water bodies. The second hierarchy encompasses 15 subclasses for the level-one categories. Figure 2 shows the level-two subclasses for Austria (13 of the 15 available classes are present). The finest scale category, level three, further sub-divides the level-two categories into the mentioned 44 finer scaled land cover classes.

The CLC classifications are based on computer-assisted visual interpretation of satellite images with a target scale of 1:100.000 and a minimum mapping unit of 25 ha (Heymann et al. 1994)

By making use of the presented object-oriented data modeling possibilities it will be shown how a change comparison of the different layers can be improved, especially when taking into account changes between subclasses and superclasses and aggregating changes to composite objects in an integrated framework.

### Study area and data

In this study, CLC data for Austria from 2000 and 2006 were analyzed as a proof of concept (cf. Figure 2). Because the nomenclature of CLC is consistent for almost all of Europe, the approach is completely transferable to other CLC data sets.

The CLC data from 2000 and 2006 were obtained as shapefiles (from the European Environment agency, EEA; http://www.eea.europa.eu), with the geometry is reflecting the finest level-three category, and the classification of the different levels accessible through attribute tables.

### Implementation

**Figure 3.**
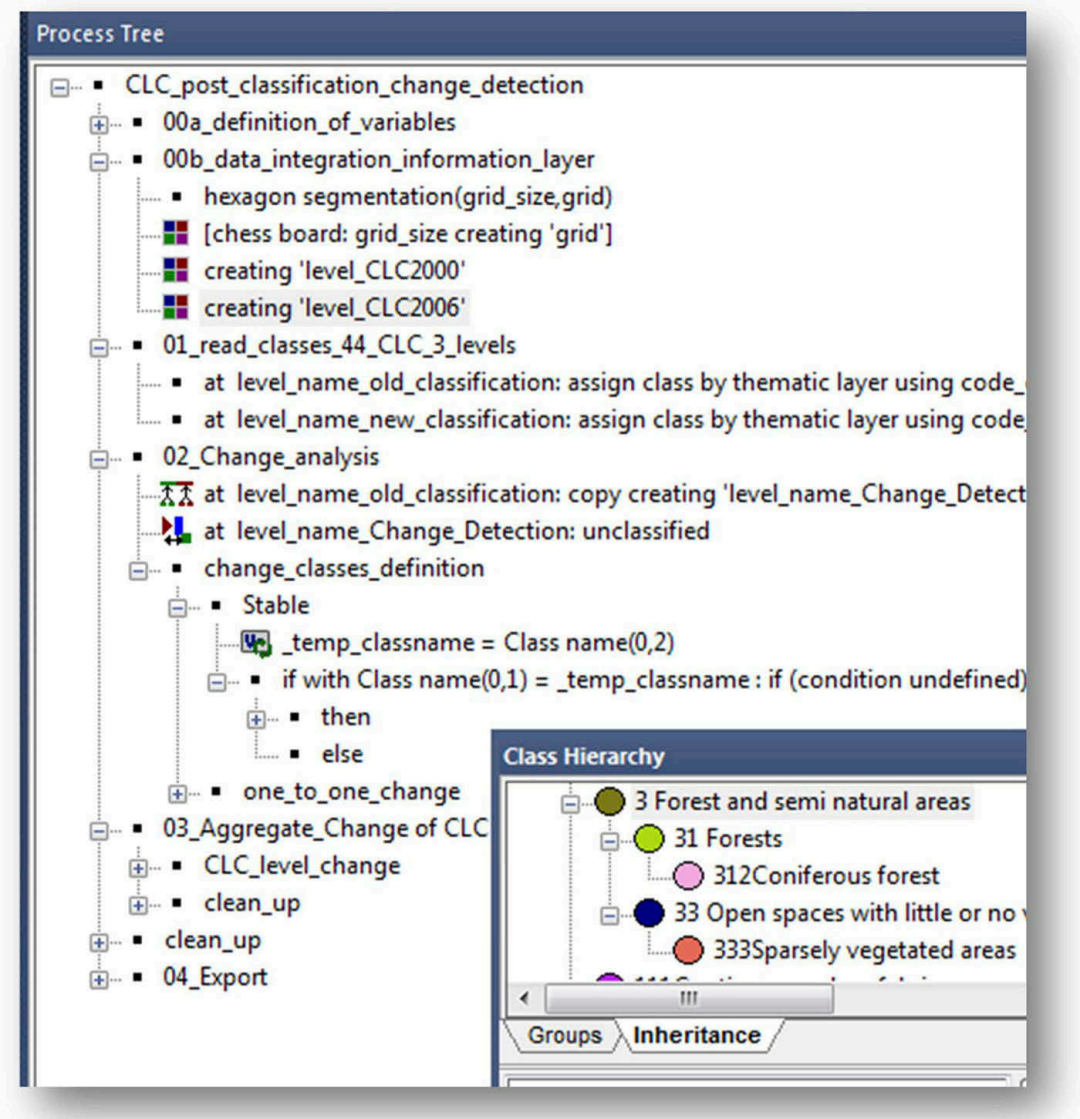
Screenshot of a subset of the rule-set programmed in CNL for a fully automated change comparison of CLC data sets. On the lower right, an example of the class hierarchy is shown where the CLC hierarchical classification system can be realized (including inheritance of properties/rules).

An object-by-object change comparison framework was set up within eCognition. This was accomplished using a modular programming language (cognition network language (CNL)) which controls the process of import routines and analytical steps and can also handle object-specific queries. A rule-set is created which can be saved (like a protocol, see Figure 3), and once set up and parameterized, the calculation steps are all automated and transferable. The existing data layers were imported into that framework, and an initial class hierarchy was automatically created, based on the CLC classification attributes. Figure 3 shows (lower right) a subset of the class hierarchy where the hierarchical classification system of the CLC can be realized (including inheritance). This grouping of classes is comparable to the object-oriented data modeling *concept of generalization*, and the advantage is that not only data inherent hierarchies can be set up, but also new groupings, for example, regarding certain change classes, are realizable with a simple drag and drop (cf. Figure 1). In addition, applying the object-oriented concept of generalization improves the change comparison of data if, for instance, different land cover nomenclatures are used for different time slots, which often causes problems in PCCs (cf. Ahlqvist 2008; Robbins and Maddock 2000). As a result, different nomenclatures can be regrouped in common classes for a change comparison without changing the original class names.

**Figure 4.**
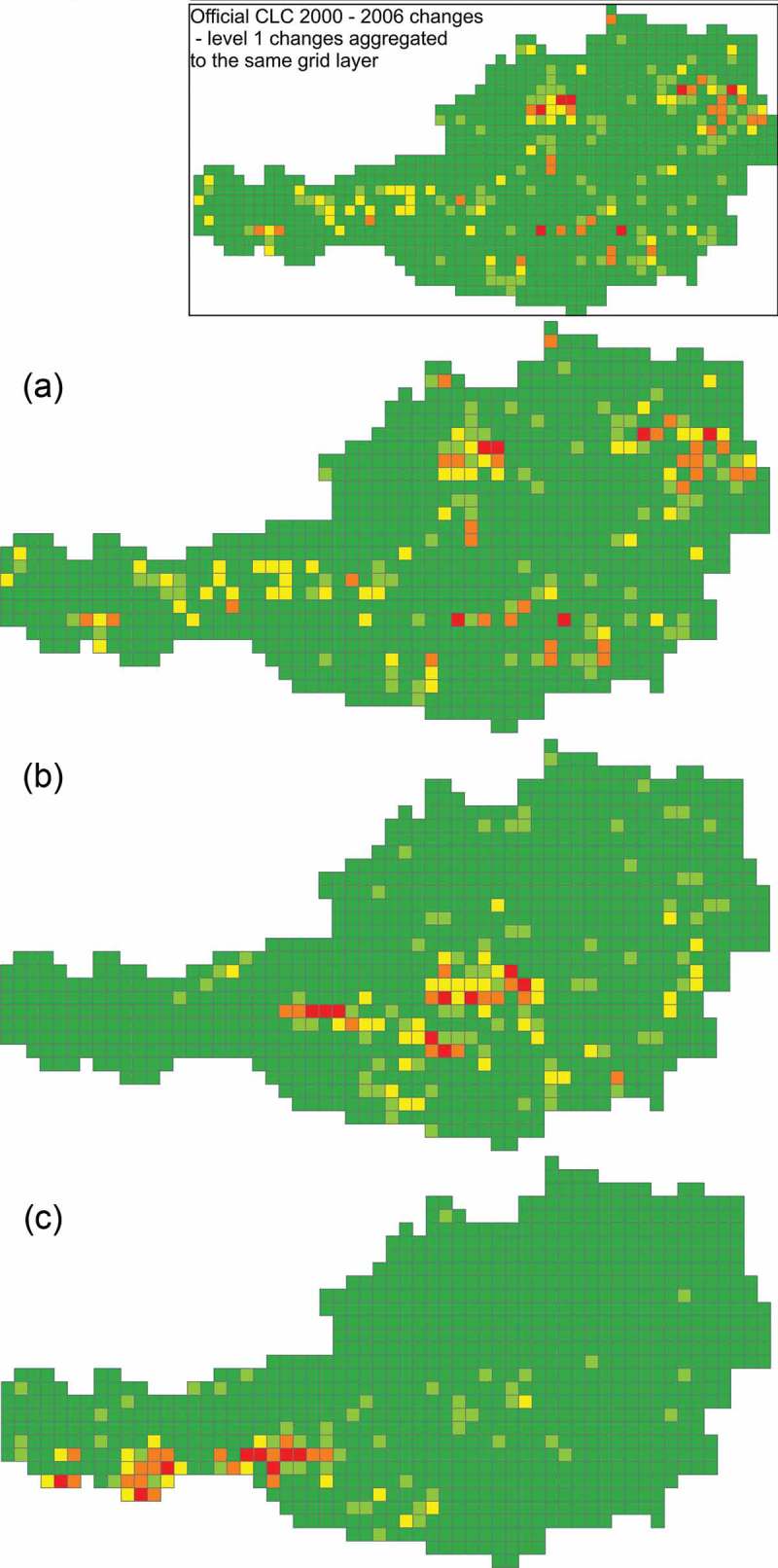
CLC change comparison for 2000 and 2006: (a) changes between CLC classes of level one, (b) changes between CLC classes of level two, and (c) changes between CLC classes of level three. The red tones indicate a high amount of change per 10 × 10 km grid cell, dark green tones represent minor or no change. The uppermost figure depicts the official CLC 2000–2006 changes for level-one changes, aggregated to the same grid layer.

The change comparison is performed on different hierarchical levels: the highest level is defined as a reporting unit layer (change-detection layer), the two CLC time slots (here: 2000 and 2006), and the lowest level is a complete intersect (similar to an overlay operation in a GIS, see also Figure 1). The reporting unit layer’s geometry is flexible according to the specific research question, which addresses the object-oriented data modeling *concept of aggregation*. Different geometries for the reporting units are demonstrated in Figures 4 and 6, including regular geometry units such as grids or hexagons as well as administrative units.

**Figure 5.**
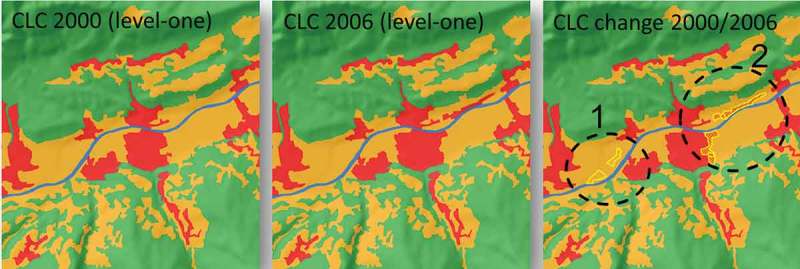
Level-one CLC classes for the years 2000 and 2006. Official changes for the class “artificial surfaces” are shown on the most right figure, with yellow outlines. Circle 1 highlights changes which were not present in the original CLC layers but were later introduced by manual interpretation based on images. Circle 2 shows changes present in the original CLC layers, and which were therefore detected by the presented overlay method.

The reporting units provide information about the changes in the underlying hierarchy. Because the object-model is topological (in horizontal direction between objects of the same layer) and also vertical between the different layers), relationships between the CLC 2000 and 2006 layers can be queried on the reporting units. Figure 4 shows the relative area of change between different CLC categories data layers investigated; theoretically, a plethora of additional information could be handled here. Figure 5(a) shows, for example, the change of the level-three class “335 Glaciers and perpetual snow” into another class (here: aggregated to hexagon units). This flexibility in reporting relationships between objects is reflecting the third object-oriented data modeling concept, the *concept of association*. In addition, topological relationships can be used to perform certain “clean-up” routines. For example, sliver polygons can be merged with the largest neighbor, or the neighbor with the longest border, or the neighbor with the most similar class of the same subcategory, etc.

## Results and discussion

**Figure 6.**
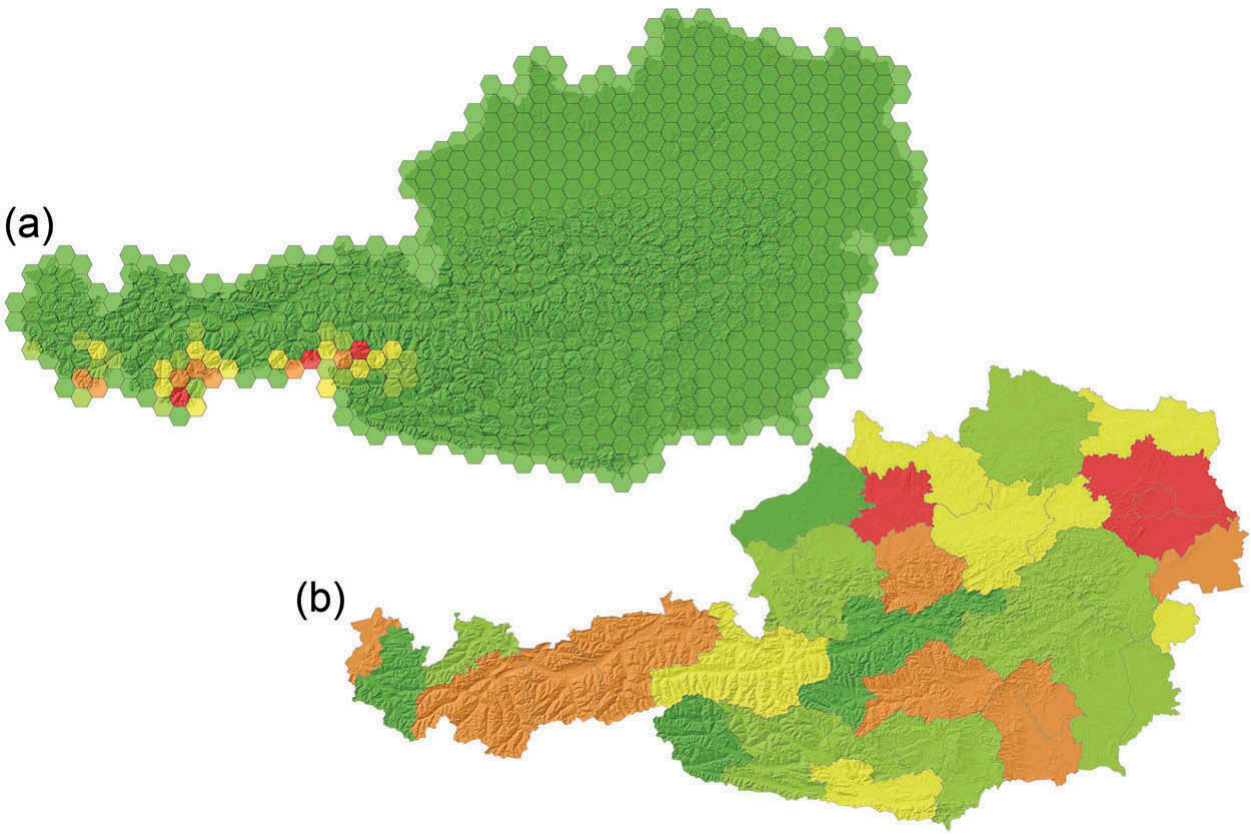
CLC change comparison of 2000/2006 aggregated to different reporting units for changes between CLC classes: (a) change of the level-three class “335 Glaciers and perpetual snow” into another class aggregated to hexagons, (b) changes between level-one classes aggregated to administrative The Nomenclature of Territorial Units for Statistics (NUTS) 3 units in Austria. Red tones indicate a higher amount of change per aggregation unit; dark green tones show minor changes or no change.

Three aggregated change classes were defined and calculated, reflecting changes between the classes of the three-level CORINE nomenclature: (1) changes between CLC level-one classes (5 main classes), (2) changes between CLC level-two classes within level-one super classes (15 subclasses), and (3) CLC level-three changes within level-two super classes (44 classes on this most detailed level). These aggregated classes were then automatically calculated by programming specific rules which parse and assign the triple-digit CLC class numbers to the relevant change classes. Since the class legend is a hierarchy including inheritance, the classes were assigned as child classes to the parent-change classes. This enables the user to easily change specific allocations, if needed, by a simple drag and drop approach. The changes were automatically reflected in the change-aggregation layer. Figure 4 shows the results for the area of Austria, with different CLC class level changes. The changes were aggregated to a regular gridded layer (10 × 10 km); this gave a good overview of spatial change patterns, which could be examined further in detail. For instance, the marked changes between level-one classes applied mainly to denser populated/urban regions, whereas level-three changes (within the level-two superclasses) were mainly observed in the higher Alpine regions (mostly changes of the class “Glaciers and perpetual snow”, see also Figure 6(a)).

The change-aggregation layer calculated from the CLC 2000/2006 change comparison and the official CLC change polygons (aggregated to the same grid, see Figure 4) for level-one changes revealed an almost perfect agreement. This was expected, since the change comparison is based on the same data, and the comparison in this example is strictly deterministic. Nevertheless, there were some small differences. The reason is that the official CLC change polygons are manually derived from an expert interpretation, by comparing 2000 and 2006 image data in which also a revision of errors – potentially present in the CLC2000/2006 data – was conducted. In addition, the expert interpretation of changes could lead to variations “from the codes occurring on the CLC2000 map and/or in the final CLC2006 map due to generalization applied in producing CLC2000 and CLC2006” (cf. Büttner et al. 2012; Feranec et al. 2007). Figure 5 shows an example of changes not reflected in the original CLC layers but introduced in the expert interpretation of changes.

The implemented CNL rule-set is completely transferable to every CLC 2000/2006 data set in Europe; it is automatically applicable if an analysis of the same change classes is requested. The size and form of the grids (reporting units) can be changed easily, as can the target change classes and the hierarchical information of the change comparison framework per grid unit: Figure 6(a) shows results of an analysis of changes of the level-three class “335 Glaciers and perpetual snow” into another class aggregated to hexagon units. Figure 6(b) shows level-one changes as demonstrated in Figure 4 but aggregated to NUTS units, a geocode standard for referencing the administrative divisions of countries for statistical purposes (the 35 units in NUTS level three consist of merged municipalities).

## Conclusion

Selected concepts of object-oriented data modeling were shown to improve PCC analysis in terms of flexibility, transferability, and ease of use. Because the concepts of generalization, association, and aggregation are not available in conventional GIS, this research investigated a methodology for utilizing OBIA techniques in GIS operations. The approach is programmed in a repeatable rule-set, allowing the analysis of any CLC layer comparison in Europe. But the approach is not limited to CLC layers; with minor modifications any change comparison of different polygon or raster layers is feasible. Even the comparison of more than two time stamps is possible, because the object-hierarchy is not limited to a certain number of layers.

Because of the original purpose of the eCognition software to analyze imagery, the data model has some limitations: First, only polygon vector or raster data is supported. Second, the topologically enabled multi-scale hierarchy builds upon pixels as smallest units. This is not only due to the original purpose of the software, but also a concession with regard to the computing power needed. Even with the possibility to reduce the pixel size to a minimum – imported vector data is always converted into “rasterized” vector data, which has to be taken into account for a productive use of the presented methodology. The purpose of using the proposed methodology would be defeated if, for instance, high-precision output geometry was needed as a result of the change comparison. But if the change comparison should result in flexible, transferable, and highly complex change comparisons that can be visualized or calculated and aggregated to higher level composite objects (reporting units), then the approach proposed here offers a new avenue of tackling these tasks compared to conventional GIS.
